# The Book World of Medicine and Science

**Published:** 1899-06-17

**Authors:** 


### The Book World of Medicine and Science.

The Use of Tuberculin for Lessening the Prevalence of Tuberculosis. Pamphlet, by Harold Scurfield, M.D. (H. and W. Brown, 30, Fulham Road, S.W.)

Dr. Scurfield's tersely-written pamphlet may be recommended as conveying the salient points of our present information on the question. It has long since passed out of the region of probability into that of certainty that cows having tuberculosis produce milk containing the tubercle bacillus; that this infected milk possesses extraordinary virulence, and is a cause, and a frequent cause, of tuberculous disease in children. Until the public refuse to consume milk from any dairy not using the tuberculin test, it will be encouraging the spread of disease, for we much doubt whether the average farmer will ever employ this simple test unless he can be got to see that it his interest so to do, or unless the test be made compulsory.

Hygiene and Public Health. By B. Arthur Whitelegge, M.D., B.Sc.Lond., F.R.C.P., D.P.H.Camb. Seventh thousand. (London: Cassell and Co. 1899. Price 7s. 6d.)

Although it is not necessary to say much about a book the repeated issues of which continually testify to its popularity, we must give a word of welcome to this the seventh thousand of one of the most useful of all handbooks of hygiene. In view of the great bulk of some of the volumes which it is our fate to have to consult from time to time on subjects connected with hygiene, it has often been to us a matter of interest and, indeed, of surprise, to note how fully Dr. Whitelegge has been able to squeeze into this little work all that the practitioner really wants to know. In so progressive a science as that of hygiene, however, no book written ten years ago can satisfy the requirements of the practitioner of to-day, and it may truly be reckoned as one of the virtues of small books that without great loss they can be replaced as fresh years bring forth fresh facts, fresh theories, and especially, in regard to this subject, fresh laws. Hence the advantage of a handbook which for the moderate outlay of 7s. 6d. gives a fair survey of, and at the very least serves as a most useful index to, the present state of our knowledge in regard to matters of public health. The principal changes in this edition have to do with recent developments in regard to vital statistics, etiology, disinfection, sewage disposal, and vaccination. Under the head of disinfection the newer disinfectants such as formic aldehyde vapour are dealt with, as well as the various forms of apparatus for disinfection by heat; while in the chapters on the infectious diseases recognition is given to the more recent developments of bacteriology, a short outline is presented of the provisions of the Vaccination Act of last year, and reference is made to the diffusion of diphtheria by schools and to the relation between abnormal prevalences of enteric fever in towns supplied by river water and the existence of floods above the "intakes" of their water supplies. Recent observations in regard to malaria also are referred to. On the other hand, as showing how impossible it is to be up-to-date all round, it must be pointed out that almost coincidently with the publication of this book a new edition of the Code of Rules for the Prevention of Infectious and Contagious Diseases has been issued by the Association of Medical Officers of Schools, which throws somewhat out of date the quotations from it which are given as to the periods of quarantine, &c., to be observed after attacks of infectious disease. Under the head of sewage disposal various recently-introduced methods are mentioned. We are a little surprised that the various plans which are now on their trial for the biological treatment of sewage are not dealt with at greater length than they are. Perhaps the very fact that they are still on trial has something to do with this, for the reader will soon notice, as he looks over this book, that it tends to deal with things proved rather than with things under discussion, and that where there are two sides to a question it is generally the official view that is endorsed. This is only natural in the work of one who has held high office in the sanitary service, and we may say also that it adds much to the utility of the book as an authoritative exposition of the official view of sanitary science, for, although this may not always be correct, it is an aspect of the matter with which every medical officer must needs be thoroughly familiar.

The Construction of Hospitals for Consumption and other Infectious Diseases. By John W. Hayward, M.D. (Liverpool: Egerton Smith and Co. Pamphlet, pp. 36. Price 1s.)

There is much in this pamphlet which may be very warmly commended. For instance, the remarks on materials for construction, on the absence of basements except for ventilation, on the absence of corners and angles, on the necessity for a layer of concrete or asphalt on the whole of the ground space, and generally what is said about the aspect of wards and the principles of ventilation may be freely accepted, although we hardly agree with Dr. Hayward that the tops of the windows should be eighteen inches from the ceiling. They should be as near the ceiling as possible, and they should be double, so that the lower sash of the outer window and the upper sash of the inner one may be left open. But it is chiefly with Dr. Hayward's ideas of warming hospitals that we are reluctantly compelled to dissent, unless, indeed, his hospitals are intended only as comfortable residences during the last few months of life. We do not, however, gather that this is the case. The winter temperature of the wards is to be 65 deg., and, except in small rooms, it is to be maintained by hot air or by steam pipes. Now we believe that the practice of warming our bodies with the air we breathe is as injurious as it is unnatural. The rays from an open fire resemble those from the sun, and pass through the air without appreciably warming it, whereas all artificial systems really warm the air, and they should not be used unless it can be proved that they are the lesser of two evils. That air of almost any degree of coldness may be inhaled with benefit by consumptives, if only the surface of the body be kept warm by sufficient clothing, seems proved beyond the possibility of cavil, by the satisfactory results obtained in the German consumption hospitals. At these the patients spend fourteen hours a day out-of-doors, generally reclining on sofas, winter and summer. Their bedroom windows are invariably open during the night, and Dr. Hayward's maxim, that "the admission of cold air is particularly injurious during sleep," would meet with scant agreement by those German physicians who are devoting their whole lives to the study and treatment of consumption.

